# Longitudinal surveillance of influenza in Japan, 2006–2016

**DOI:** 10.1038/s41598-022-15867-3

**Published:** 2022-07-14

**Authors:** Shinako Inaida, Shigeo Matsuno, Jiro Okumura

**Affiliations:** 1 Graduate School of Medicine, Kyoto University, Yoshidakonoecho, Sakyo-ku, Kyoto, 606-8501 Japan; 2 Department of Environmental Medicine and Behavioral Science, Kindai University Faculty of Medicine, Osakasayama, Japan; 3 Biomedical Science Association, Tokyo, Japan

**Keywords:** Medical research, Diseases, Infectious diseases, Influenza virus, Influenza virus, Viral infection

## Abstract

We analysed 2006–2016 national influenza surveillance data in Japan with regards to age-, sex-, and predominant virus-related epidemic patterns and the prevalence of serum influenza virus antibodies. We found a significant increase in influenza prevalence in both children (≤ 19 years old) and adults (≥ 20 years old) over time. The influenza prevalence was higher in children (0.33 [95% CI 0.26–0.40]) than in adults (0.09 [95% CI 0.07–0.11]). Additionally, the mean prevalence of antibodies for A(H1N1)pdm09 and A(H3N2) was significantly higher in children than in adults, whereas the mean prevalence of antibodies for B lineages was relatively low in both children and adults. There was a biennial cycle of the epidemic peak in children, which was associated with a relatively higher prevalence of B lineages. The female-to-male ratios of the influenza prevalence were significantly different in children (≤ 19 years old; 1.10 [95% CI:1.08–1.13]), adults (20–59 years old; 0.79 [95% CI 0.75–0.82]), and older adults (≥ 60 years old; 1.01 [95% CI 0.97–1.04]). The significant increase in influenza prevalence throughout the study period suggests a change of immunity to influenza infection. Long-term surveillance is important for developing a strategy to monitor, prevent and control for influenza epidemics.

## Introduction

Seasonal influenza epidemics occur every winter in mild temperate regions^1–4^. In Japan, the usual influenza epidemic starts around the beginning of January and peaks within a few weeks^5–8^. The influenza epidemic curve in Japan shows exponential growth toward the peak week and immediately decreases thereafter. This recurrent pattern has been also found in other respiratory viruses, such as the COVID-19 epidemic, in winter^9^. Furthermore, the spread of the epidemic is similarly located in regions over time for different predominant influenza viruses^8^. For example, during the pandemic caused by the novel swine influenza variant A(H1N1)pdm in 2009, epidemic clustering and transmission similar to that of seasonal influenza was observed^8,10–17^. This event highlighted the importance of understanding epidemic trends to estimate the risk of an epidemic. Similarly, continuous and long-term surveillance is important for the prevention of and preparation against emerging and re-emerging influenza epidemics^5,18^. In Japan, influenza surveillance has been conducted across the country by local health centres and public health institutes and infectious disease surveillance centres of the prefectural governments under the guidelines of the Ministry of Health, Labour and Welfare. Surveillance was widely expanded in the 1980s, with an increased number of sentinel clinics that report the weekly number of outpatient incident cases^5^.

In this study, we analyzed the recent trends of influenza epidemics, including the age- and sex-related prevalence and the seasonal predominant virus with respect to the prevalence of influenza antibodies, using national surveillance data obtained between 2006 and 2016 in Japan.

## Methods

### Surveillance data

We used national influenza surveillance data for Japan from 2006 to 2016, thereby covering a period spanning from the 2005–2006 influenza season to the 2015–2016 influenza season^5^. The surveillance data were collected at 5000 sentinel clinics across Japan (approximately 3000 paediatric clinics and 2000 clinics of general practitioners for adults)^5^. The number of sentinel clinics was decided according to the population size of the administrative sector of the local health centres, making the surveillance similar nationwide. The majority of sentinel clinics have been settled, and neither the number and location of the sentinel clinics in the surveillance system nor the estimated coverage of the population by the sentinel clinics changed much over this time. In addition, the population coverage of sentinel clinics was similar for children and adults (from the estimated total population coverage by sentinel clinics and calculated from the coverage percentage of sentinel clinics within all medical clinics in Japan [Supplementary Table S1]).

During influenza surveillance, physicians report incident cases to the local health centre via fax according to the clinical diagnosis for influenza-like illnesses (ILIs). The diagnosis of ILI by a physician is usually made using a rapid diagnostic testing kit for patients who presented with any of the four major symptoms of ILI (high fever, sudden illness onset, upper respiratory inflammation, and generalized symptoms such as malaise). Local health centres report the weekly number of incident cases to the prefectural public health institutes or infectious disease surveillance centres, which are responsible for entering the incident cases (sex and age group [0–5 months, 6–11 months, and 1, 2, 3, 4, 5, 6, 7, 8, 9, 10–14, 15–19, 20–29, 30–39, 40–59, and over 60 years of age]) in the online database of the National Epidemiological Surveillance of Infectious Diseases (NESID), National Institute of Infectious Diseases (NIID)^6^. The epidemiologic period spans from July to June (comprising the influenza epidemic period of January to March). The NESID data are available online on the NIID website^6^. For the observed timeframe, pathogen surveillance was conducted using polymerase chain reactions (PCR)^7^ of nasal swab specimens collected in approximately 10% of patients with ILI across the country. PCR for virus testing were conducted at the prefectural public health institute or NIID, and the results were uploaded onto the NIID website^6,7^.

Separately, surveillance for serum antibodies against each influenza virus subtype and strain was conducted at designated hospitals across the country using the hemagglutination inhibition (HAI) assay^19^. Samples of the serology surveillance were randomly collected from approximately 6000 Japanese individuals each year. We compared the influenza prevalence rate and the prevalence rate of serum influenza virus antibody.

Statistical analysis was performed using IBM SPSS version 21 (IBM Corp., Armonk, NY, USA). The level of significance was set to 5%.

### Time-series analysis of the influenza prevalence rate and predominant virus

We calculated the yearly influenza prevalence rate, which is the rate of total ILI cases within the estimated coverage population by sentinel clinics, for each age group (0–5, 6–14, 15–19, 20–29, 30–39, 40–59, and ≥60 years). The estimated coverage population by sentinel clinics was calculated for each age group using the national census data in 2010 as the coverage percentage of sentinel clinics within the total medical clinics (paediatric clinics for estimating the sentinel population coverage of children [≤ 19 years of age] and clinics of general practitioners for adults for estimating the sentinel population coverage of adults [≥ 20 years of age], Supplementary Table S1). To assess the time-series trends of the prevalence rate, we fitted a linear regression model for the influenza prevalence rate and time (season) in children (≤ 19 years of age) and adults (≥ 20 years of age).

We compared the influenza prevalence with the predominant epidemic influenza virus. We calculated the prevalence rate of each epidemic influenza virus within the total number of isolates and used Pearson’s correlation coefficient to assess the association between the influenza prevalence rate and the prevalence of each virus subtype and lineage. After observing the low- and high-prevalence seasons, we compared the influenza prevalence rate and the prevalence of each virus subtype and strain between these seasons using non-paired *t*-tests.

### Influenza antibody trends

Most samples in the serology surveillance for influenza antibody testing were collected between July and September each year to assess the results before vaccination for the epidemic season. For example, in 2009, approximately 75% of samples were collected between July and September, 15% were collected between April and June, and 10% were collected between October and December. This surveillance was carried out to estimate the epidemic level in the upcoming epidemic season. Thus, by using the predicted predominant virus strain in the upcoming epidemic season (which was also selected for the vaccine virus for the upcoming epidemic season), influenza antibody testing was implemented. (Of note, influenza vaccinations were conducted starting in November each year including 2009.) The HAI assay was conducted at all 47 prefectural public health institutes or the NIID, and the results were uploaded onto the NIID website^6^.

The virus subtypes and strains for the HAI assay were selected according to the vaccine virus subtypes and strains for the year: A(H1N1) subtype [A(H1N1)pdm09 subtype from 2009 onwards], A(H3N2) subtype, and the B lineage of the selected vaccine subtypes and strains, plus another B lineage that was not selected for the vaccine. We calculated the average prevalence with 95% confidence intervals (CIs) for an influenza antibody titer ≥ 1:40, which is considered sufficient to prevent infection^19^, by age group using the data of serology surveillance between 2006 and 2016^6,7^ for five influenza virus: A(H1N1), A(H1N1)pdm09, A(H3N2), B/Yamagata-lineage, and B/Victoria-lineage. The difference in the prevalence of influenza antibody between children (≤ 19 years old) and adults (≥ 20 years old) was assessed using paired *t*-tests for each influenza virus. For multiple comparisons, the *P* value was corrected by Bonferroni’s correction. To assess the relationship between the previous infection and the prevalence of antibodies, Pearson’s correlation between the prevalence rate of the predominant virus strain and the prevalence of antibodies in children and adults was calculated separately.

### Sex ratio of the influenza prevalence rate

We compared the sex ratio (female-to-male ratio) of the influenza prevalence rate by age groups. We also compared the difference in sex ratios among children (≤ 19 years old), adults (20–59 years old), and older adults (≥ 60 years old) using a one-way ANOVA. For multiple comparisons, the P value was corrected using Bonferroni’s correction.

### Ethical approval

All methods were performed in accordance with relevant guidelines and regulations.

## Results

### Influenza prevalence and predominant virus

**Figure 1:**
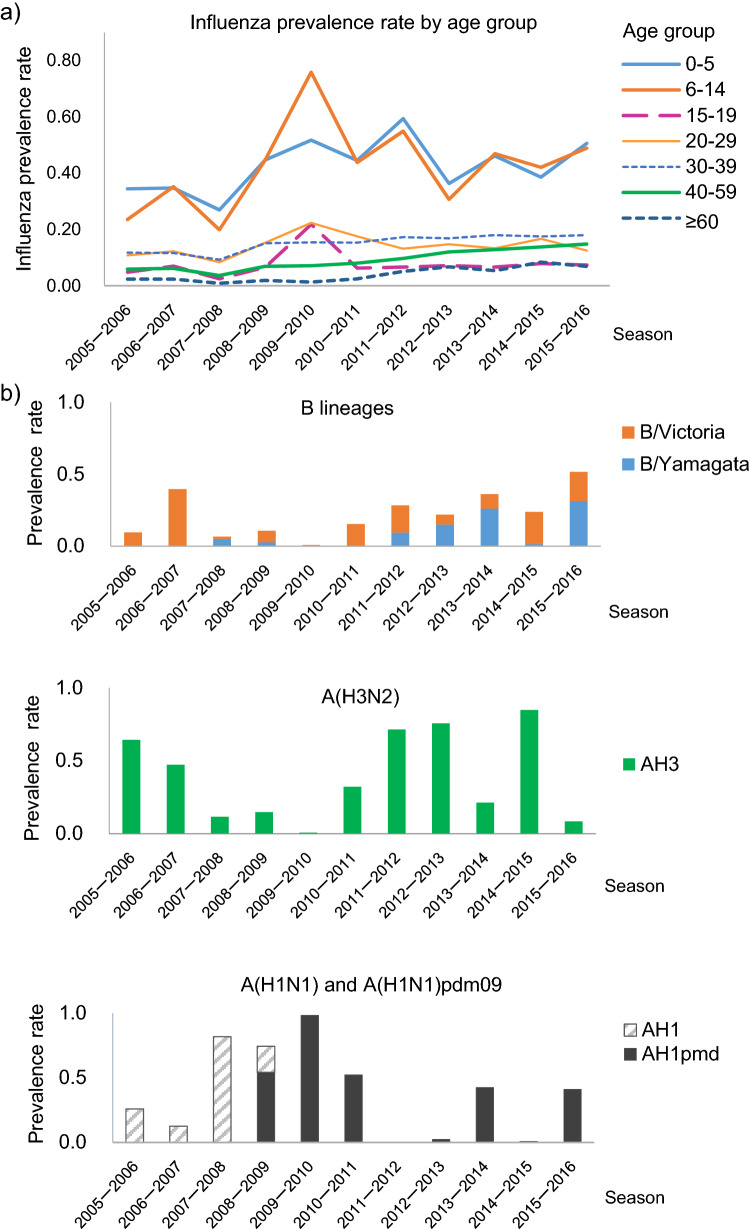
Influenza prevalence rate by age group and predominant virus. (**a**) Prevalence rate by age group according to reports from national sentinel clinics for each season. The base population was calculated using data from the national census in 2010. The 2005–2006 season comprised only data between January and June 2006 (due to data availability). (**b**) Prevalence of cases attributable to each epidemic virus subtype and strain (concerning B lineages, the prevalence of unspecified B lineages was distributed for the ratio of the B/Victoria and B/Yamagata lineages in each season).

The influenza prevalence rate was relatively higher in children aged 0–5 and 6–14 years old. The influenza prevalence rate in children younger than 20 years old was highest during the 2009–2010 season with the emergence of the novel swine influenza virus A(H1N1)pdm09 (Fig. 1a,b). There was no obvious peak of the influenza prevalence rate observed in age groups older than 30 years old during the 2009–2010 season. The age group 20–29 years old exhibited a relatively small increase in the influenza prevalence rate in the 2009–2010 season (Fig. 1a). The average influenza prevalence rate was 0.33 (95% CI 0.26–0.40) in children (≤ 19 years old) and 0.09 (95% CI 0.07–0.11) in adults (≥ 20 years old). (The number of raw incident cases by year is shown in Supplementary Table S2).

**Figure 2:**
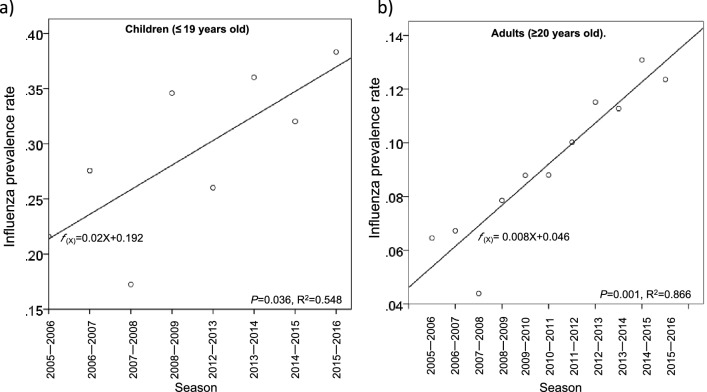
Linear regression model for seasonal influenza prevalence rate. A single linear regression model for the seasonal influenza prevalence rate in (**a**) children (≤ 19 years old) and (**b**) adults (≥ 20 years old). *f(x)* represents the prevalence rate, and X represents the number of seasons after the 2004–2005 season. For children, the seasons in which A(H1N1)pdm09 emerged (between the 2009–2010 and 2011–2012 seasons) were excluded from the regression model.

The influenza prevalence rate gradually increased over time in both children and adults (Fig. 2a,b, and Supplementary Tables S3 ). In a linear regression model, the relationship between the influenza prevalence rate and time (season) was significant in both children (*P *= 0.036) and adults (*P* = 0.001; Fig. 2a,b).

After the 2009–2010 season, the A(H1N1) subtype was replaced by the A(H1N1)pdm09 subtype, which became the predominant strain almost biennially (Fig. 1b, and Supplementary Table S4). The prevalence of the A(H1N1) subtype (and the A(H1N1)pmd09 subtype from 2009 onwards) was relatively high when that of the A/H3N2 subtype was relatively low (Fig. 1b, and Supplementary Table S4). A significant negative correlation was observed between the prevalence rates of A(H1N1) and A(H1N1)pmd09 (2009 onwards) and of A(H3N2) (r =  − 0.89, *P* < 0.01; Fig. 1b, and Supplementary Table S4).

**Figure 3:**
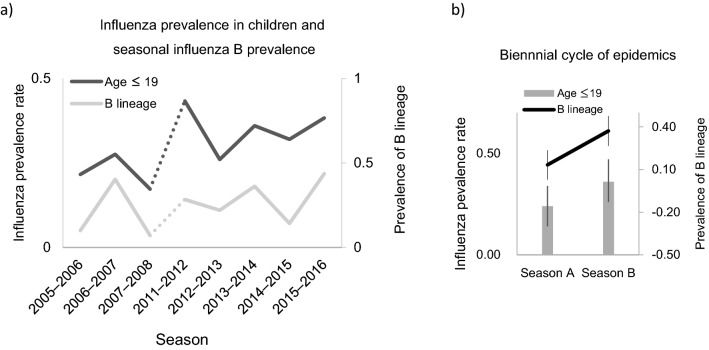
Biennial cycle of influenza virus B lineages and influenza prevalence rate in children (≤ 19 years old). (**a**) Biennial cycle of increased influenza prevalence rates in children and the prevalence of B lineages (the data exclude epidemic seasons during which the novel swine influenza virus emerged [between the 2009–2010 and 2011–2012 seasons], as indicated by the dotted lines). (**b**) Average influenza prevalence rate in children and average prevalence of B lineages for the low-prevalence period (Season A: 2005–2006, 2007–2008, 2012–2013, and 2014–2015 seasons) and high-prevalence period (Season B: 2006–2007, 2011–2012, 2013–2014, and 2015–2016 seasons).

Among the B lineages, B/Victoria was predominant over the B/Yamagata in most seasons (except the 2007–2008, 2012–2013, 2013–2014, and 2015–2016 seasons). The prevalence of the B/Yamagata-lineage increased gradually in most recent seasons. Epidemic peaks in the influenza prevalence rate in children appeared biennially, except for the 2009–2010 season (Fig. 1a, Supplementary Table S4). This biennial peak of the influenza prevalence rate in children coincided with an increase in the prevalence of influenza B lineages (B/Victoria and B/Yamagata lineages, excluding epidemic seasons with emergence of the novel swine flu virus [between the 2009–2010 and 2011–2012 seasons]; Fig. 3a). There was a significant positive correlation between the influenza prevalence rates in children and the prevalence of the B lineages (r = 0.66, *P* < 0.05, excluding epidemic seasons with emergence of the novel swine flu virus [between the 2009–2010 and 2011–2012 seasons]). In children, the influenza prevalence rate increased significantly during high-prevalence seasons (50%, *P *= 0.038; Fig. 3b) in association with a significant increase (185%, *P* = 0.002; Fig. 3b) in the prevalence of B lineages compared with the results in low-prevalence seasons. There was no biennial peak in the influenza prevalence in adults (≥ 20 years of age; Fig. 1a).

### Influenza antibody prevalence

**Figure 4:**
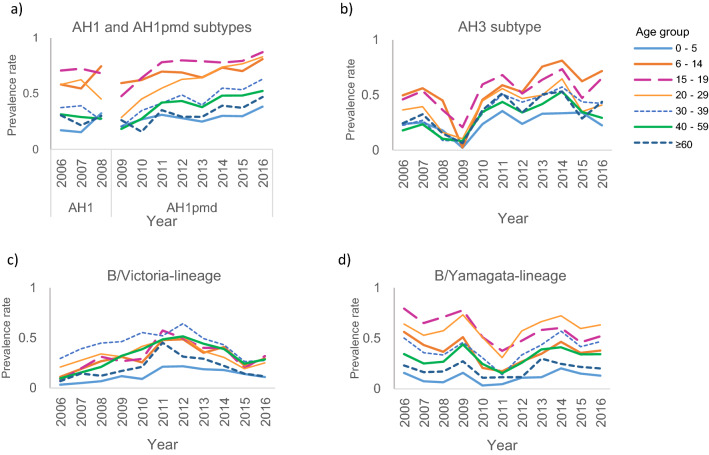
Yearly prevalence rate of hemagglutination inhibition (HAI) titers (≥ 1:40) for the influenza virus by age group. Prevalence rate of serum antibodies against influenza virus strains by age group presented for titers ≥ 1:40, as measured in approximately 6000 people in Japan each year using the HAI assay. In (**a**), the prevalence rate of serum antibodies against A(H1N1) is presented for years between 2006 and 2008, and the prevalence rate of serum antibodies against A(H1N1)pdm09 is presented for years between 2009 and 2016, although the subtypes of A(H1N1) and A(H1N1)pdm09 were genetically different.

Overall, in all age groups, the prevalence rates of serum antibody (HAI titers ≥ 1:40) did not largely change over time (Fig. 4). The prevalence rates of serum antibodies were relatively low in children 0–5 years old for all influenza A subtypes and B lineages. The mean prevalence rates of antibodies against both A(H1N1) and A(H1N1)pdm09 were relatively higher in the age groups of 6–14, 15–19, and 20–29 years old (Fig. 4). Similarly, the prevalence rates of antibodies against the A/H3N2 subtype were relatively higher in the age groups of 6–14, 15–19, and 20–29 years old. The prevalence of antibodies against A(H1N1)pdm09 and A(H3N2) increased slightly in most age groups over time. For the B/Victoria-lineage, all age groups displayed a relatively low prevalence of antibodies over time. The prevalence of antibodies against the B/Yamagata-lineage was relatively higher in the age groups of 15–19 and 20–29 years old but not in those of 6–14 years old (mean prevalence rate, 0.37 [95% CI 0.29–0.45]) or in other age groups (Fig. 4). Supplementary Table S5 lists the vaccine virus used for the HAI assay.

On average, the mean prevalence of antibodies for A(H1N1)pdm09 was significantly higher (*P* < 0.001) in children (0.57 [95% CI 0.51–0.64]) than in adults (0.45 [95% CI 0.34–0.55]; Supplementary Fig. S1, and Table S6), as too was the mean prevalence of antibodies for A(H3N2) (*P* = 0.001; in children: 0.44 [95% CI 0.35–0.54]; in adults: 0.35 [95% CI 0.25–0.45]). The mean prevalence of antibodies for B/Victoria-lineage was significantly lower (*P* = 0.001) in children (0.25 [95% CI 0.18–0.32]) than in adults (0.32 [95% CI 0.25–0.40]) (Supplementary Fig. S1). For the B/Yamagata-lineage, the mean prevalence of antibodies was similar (*P* = 0.391) between children (0.36 [95% CI 0.30–0.42]) and adults (0.37 [95% CI 0.31–0.44]). There was no correlation between the prevalence rate of the predominant virus strain and the prevalence of antibodies in children or adults.

### Sex ratio of the influenza prevalence rate according to age group

**Figure 5:**
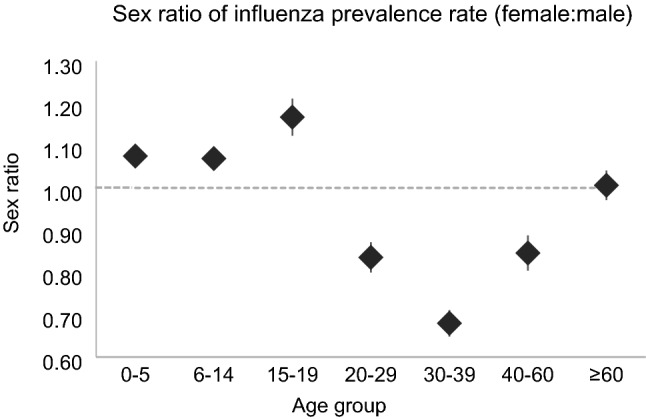
Sex ratio of the influenza prevalence rate by age group. Average female-to-male ratio in the influenza prevalence rate in each age group.

The female-to-male ratio of the influenza prevalence was consistent over time. Figure 5 presents the average sex ratio for the influenza prevalence rate, and Supplementary Fig. S2 presents the influenza prevalence rate by season, sex, and age group.

In children, the difference of the influenza prevalence rate between girls and boys was largest in those aged 15–19 years (1.17 [95% CI 1.12–1.21]). In adults, the difference of the influenza prevalence rate between women and men was largest in those aged 30–39 years (0.68 [95% CI 0.65–0.71]; Fig. 5). On average, the sex ratio for the influenza prevalence rate was opposite between children and adults; specifically, the sex ratio for the influenza prevalence rate was 1.10 (95% CI 1.08–1.13) in children (≤ 19 years of age) and 0.79 (95% CI 0.75–0.82) in adults (20–59 years of age). In older adults (≥ 60 years old), there was no difference in the influenza prevalence rate between women and men (1.01 [95% CI 0.97–1.04]). The sex ratios among children, adults, and older adults were significantly different according to a one-way ANOVA (children vs. adults: *P* < 0.001; children vs. older adults: *P* = 0.001; adults vs. older adults: *P* < 0.001).

## Discussion

We found that the prevalence rate of influenza in children in Japan remained relatively high compared with adults, which is in line with former studies^20^. The estimated average influenza prevalence rates were 0.33 in children and 0.09 in adults. In our recent study using claims-based data, the influenza prevalence rate in adults (≥ 30 years old) between 2005 and 2010 was 0.09 (95% CI 0.06–0.11), which was the same as that in the present study^21^. Although the predominant influenza virus varied by year, including the seasons in which the A(H1N1)pdm09 subtype emerged, the sex ratio of the influenza prevalence rate was consistent among all age groups. We also found a significant increase in the influenza prevalence in both children (≤ 19 years old) and adults (≥ 20 years old) over time. The reason for this increase is unknown; however, the results suggest a change in immunity to seasonal influenza infection. Because the mean prevalence of antibodies for B lineages was relatively low in both children and adults, one factor may be the increase of predominant B lineages, especially the B/Yamagata-lineage. Prevention with respect to these epidemic trends, such as improved vaccination, i.e. matching the vaccine strain and timeliness, as well as meeting a sufficient rate of vaccination coverage for age and sex groups with a higher rate of prevalence, should be sought^4^.

Although the serum antibody prevalence rates against the vaccine strains were relatively low in adults (except those aged 20–29 years), the influenza prevalence rate in adults was lower than in children. This observation may indicate the existence of immunity in adults from previous infection by different virus subtypes and strains^22–25^. Similarly, there was no increase in the influenza prevalence rate in adults during the emergence of the A(H1N1)pdm09 subtype, suggesting that adults may have protective immunity against this subtype. Conversely, the results suggested that children are more susceptible to emergent virus subtypes and strains. The relatively higher prevalence of antibodies against A(H1N1)pdm09 could be attributed to the timing of the sample collection for the HAI assay in 2009, which occurred after the epidemic wave of A(H1N1)pdm09 started in August^26^. Considering the timing of the serum sampling, which mostly occurred before vaccination for the upcoming epidemic season, the observed prevalence of antibodies is thought to be derived from natural infection in the previous epidemic season; the increase in the prevalence of antibodies against A(H1N1)pdm09 over time may suggest residual antibodies from previous influenza infections. Although the HAI assay was conducted for vaccine virus strains, further data on antibodies using the Enzyme-Linked Immuno Sorbent Assay and whole influenza virus for antibody testing should be considered^27^. Additionally, our data of serum influenza antibodies included no information regarding the individual vaccination status.

National surveillance data for influenza over a long period, including data for age, sex, predominant virus subtypes and strains, and serum antibodies, are relatively limited^17^. Regarding the predominant virus subtypes, it has been found in many countries that after 2009 the A(H1N1) subtype was replaced by the A(H1N1)pdm09 subtype^25–30^. Meanwhile, our study illustrated that the prevalence of serum antibodies against influenza viruses was relatively low in children (0–5 years old), including antibodies against both the B/Yamagata and B/Victoria lineages. The biennial increase in the influenza prevalence rate in children in Japan during the study period appears attributable to epidemics caused by B lineage viruses. After 2010, the B/Yamagata-lineage has predominated over the B/Victoria lineage globally (e.g., Africa, Latin America, Europe)^25,31–35^. In some countries, the number of infections by B lineage strains was increased in children^35^. Similarly, in Japan, the prevalence of the B/Yamagata-lineage has been increasing over time.

In Japan, current influenza vaccination coverage is estimated at approximately 80% in school-age children and 50% in older adults^36,37^. Despite this relatively high vaccination coverage, the prevalence rate of influenza has remained similar over time, including a relatively high influenza prevalence rate in children. Therefore, further research to match vaccine virus strains with the predominant epidemic virus strains and increase the effectiveness of vaccinations in each season is needed, particularly with respect to B lineages^36–40^.

Both the incidence surveillance and serology surveillance were based on data collected from the outpatients, and thus, the data may have reflected healthcare-seeking or vaccination behavior or accessibility to testing in children and adults. However, concerning differences in the influenza prevalence rate by age and sex, previous studies (such as those in Australia and Germany) reported findings similar to those in our study, including higher incidence rates in male children than in female children and higher incidence rates in female adults than in male adults^41–45^. Differences in incidence according to age and sex are thought to correspond to the immune status^46^. For example, women of reproductive age may have a stronger inflammatory response to infection than men of that age; thus, the rates of hospitalization for influenza infection may be higher in women than in men^46,47^.

The surveillance of all infectious diseases was consolidated in Japan over decades and operated at the level of sentinel clinics to all clinic networks based on the surveillance disease category under the Infectious Disease Law^5^. Furthermore, the surveillance network was immediately available for the active surveillance of COVID-19^48–50^. Long-term surveillance is important to develop and improve the strategy of monitoring, preventing and controlling future influenza epidemics.

## Supplementary Information

Supplementary Figures.

Supplementary Tables.

## Data Availability

The data of National Epidemiological Surveillance of Infectious Diseases (NESID) is available online. https://www.niid.go.jp/niid/en/data.html.
