# Supplementary material for: Longitudinal surveillance of influenza in Japan, 2006–2016
Source: Sci Rep. 2022 Jul 14;12:12026. doi: 10.1038/s41598-022-15867-3 (PMC9281223; doi:10.1038/s41598-022-15867-3)
Supplement: Supplementary file 1 — Supplementary Figures. [file 41598_2022_15867_MOESM1_ESM.docx]

Supplementary Figure S1. Yearly distribution of hemagglutination inhibition titers for influenza virus strains in children (≤19 years old) and adults (≥20 years old) for a) A(H1N1) and A(H1N1)pdm09, b) A/H3N2, c) B/Victoria-lineage, and d) B/Yamagata -lineage

a) b)

≤19

≥20

Age group

c) d)

Supplementary Figure S2. Seasonal influenza prevalence rates by sex and age group in a) children (≤19 years old) and b) adults (≥20 years old)

a)

Age group

b)

Age group
