# Supplementary material for: Longitudinal surveillance of influenza in Japan, 2006–2016
Source: Sci Rep. 2022 Jul 14;12:12026. doi: 10.1038/s41598-022-15867-3 (PMC9281223; doi:10.1038/s41598-022-15867-3)
Supplement: Supplementary file 2 — Supplementary Tables. [file 41598_2022_15867_MOESM2_ESM.docx]

Supplementary Table S1. Estimated population coverage by sentinel clinic for influenza incidence surveillance

|  | Total number of clinics^a^ | Estimated population per clinic | **Estimated total population coverage by sentinel clinics** (3000 Paediatric clinics, and 2000 clinics for adults) |
| --- | --- | --- | --- |
| Paediatric clinics | 22K | 1037 | 3110K |
| Clinics of GPs for adults | 63K | 1654 | 3309K |

^a^The data were reviewed from the statistics of medical clinics by the Ministry of Health, Labour, and Welfare, in 2008 [in Japanese]

<https://www.mhlw.go.jp/toukei/saikin/hw/iryosd/08/dl/03_0001.pdf>

Supplementary Table S2. Influenza incident case

| Year | 2006 | 2007 | 2008 | 2009 | 2010 | 2011 | 2012 | 2013 | 2014 | 2015 | 2016 |
| --- | --- | --- | --- | --- | --- | --- | --- | --- | --- | --- | --- |
| N | 900181 | 1212042 | 621447 | 3068082 | 268932 | 1363793 | 1676374 | 1166322 | 1743826 | 1169041 | 1751970 |

Supplementary Table S3. Influenza prevalence rate by age group

| Season | 0-5 yrs old | 6-14 yrs old | 15-19 yrs old | 20-29 yrs old | 30-39 yrs old | 40-59 yrs old | ≥60 yrs old |
| --- | --- | --- | --- | --- | --- | --- | --- |
| 2005ー2006 | 0.34 | 0.24 | 0.05 | 0.11 | 0.12 | 0.06 | 0.02 |
| 2006ー2007 | 0.35 | 0.35 | 0.07 | 0.12 | 0.12 | 0.06 | 0.02 |
| 2007ー2008 | 0.27 | 0.20 | 0.03 | 0.08 | 0.09 | 0.04 | 0.01 |
| 2008ー2009 | 0.45 | 0.45 | 0.07 | 0.15 | 0.15 | 0.07 | 0.02 |
| 2009ー2010 | 0.52 | 0.76 | 0.22 | 0.22 | 0.15 | 0.07 | 0.01 |
| 2010ー2011 | 0.45 | 0.44 | 0.06 | 0.18 | 0.15 | 0.08 | 0.03 |
| 2011ー2012 | 0.59 | 0.55 | 0.07 | 0.13 | 0.17 | 0.10 | 0.05 |
| 2012ー2013 | 0.36 | 0.31 | 0.07 | 0.15 | 0.17 | 0.12 | 0.07 |
| 2013ー2014 | 0.46 | 0.47 | 0.07 | 0.13 | 0.18 | 0.13 | 0.05 |
| 2014ー2015 | 0.39 | 0.42 | 0.08 | 0.17 | 0.18 | 0.14 | 0.08 |
| 2015ー2016 | 0.51 | 0.49 | 0.07 | 0.13 | 0.18 | 0.15 | 0.07 |

Supplementary Table S4. Seasonal prevalence of predominant virus

| Season | A(H1N1)pmd09 | A(H1N1) | A(H3N2) | B/Victoria | B/Yamagata |
| --- | --- | --- | --- | --- | --- |
| 2005ー2006 | 0 | 0.26 | 0.64 | 0.10 | 0 |
| 2006ー2007 | 0 | 0.13 | 0.48 | 0.39 | 0 |
| 2007ー2008 | 0 | 0.82 | 0.12 | 0.02 | 0.05 |
| 2008ー2009 | 0.54 | 0.20 | 0.15 | 0.08 | 0.03 |
| 2009ー2010 | 0.98 | 0 | 0.01 | 0.01 | 0 |
| 2010ー2011 | 0.52 | 0 | 0.32 | 0.15 | 0 |
| 2011ー2012 | 0 | 0 | 0.71 | 0.19 | 0.10 |
| 2012ー2013 | 0.02 | 0 | 0.76 | 0.07 | 0.15 |
| 2013ー2014 | 0.43 | 0 | 0.21 | 0.10 | 0.26 |
| 2014ー2015 | 0.01 | 0 | 0.75 | 0.22 | 0.02 |
| 2015ー2016 | 0.41 | 0 | 0.07 | 0.20 | 0.31 |

**Supplementary Table S5: Influenza A and B virus strains used for the HAI assay** ^a^

|  | Year |  |  |  |  |  |  |  |  |  |
| --- | --- | --- | --- | --- | --- | --- | --- | --- | --- | --- |
| virus | 2006 | 2007 | 2008 | 2009 | 2010 | 2011 | 2012 | 2013 | 2014 | 2015 |
| AH1pmd |  |  |  | A/California/7/2009pdm | A/California/7/2009pdm | A/California/7/2009pdm | A/California/7/2009pdm | A/California/7/2009pdm | A/California/7/2009pdm | A/California/7/2009pdm |
| AH1 | A/New Caledonia/20/99 | A/Solomon Islands/3/2006 | A/Brisbane/59/2007 |  |  |  |  |  |  |  |
| AH3 | A/Hiroshima/52/2005 | A/Hiroshima/52/2005 | A/Uruguay/716/2007 | A/Uruguay/716/2007 | A/Victoria/210/2009 | A/Victoria/210/2009 | A/Victoria/361/2011 | A/Texas/50/2012 | A/New York/39/2012 | A/Swiss/9715293/2013 |
| B/Victoria | B/Malaysia/2506/2004 | B/Malaysia/2506/2004 | B/Malaysia/2506/2004 | B/Brisbane/60/2008 | B/Brisbane/60/2008 | B/Brisbane/60/2008 | B/Brisbane/60/2008 | B/Brisbane/60/2008 | B/Brisbane/60/2008 | B/Texas/2/2013 |
| B/Yamagata | B/Shanghai/361/2002 | B/Florida/7/2004 | B/Florida/4/2006 | B/Florida/4/2006 | B/Florida/4/2006 | B/Wisconsin/1/2010 | B/Wisconsin/1/2010 | B/Massachusetts/02/2012 | B/Massachusetts/02/2012 | B/Phuket/3073/2013 |

^a^ The information was reviewed from Infectious Agents Surveillance Report (IASR), NIID [in Japanese]

<https://www.niid.go.jp/niid/ja/iasr.html>

Supplementary Table S6. Yearly prevalence rate of hemagglutination inhibition (HAI) titers (≥1:40) for the influenza virus by age group

A(H1N1) and A(H1N1)pmd09

| **Virus strain** | **A(H1N1)** |  |  | **A(H1N1)pmd09** |  |  |  |  |  |  |  |
| --- | --- | --- | --- | --- | --- | --- | --- | --- | --- | --- | --- |
| Year | **2006** | **2007** | **2008** | **2009** | **2010** | **2011** | **2012** | **2013** | **2014** | **2015** | **2016** |
| Age group |  |  |  |  |  |  |  |  |  |  |  |
| 0－5 | 0.17 | 0.15 | 0.32 | 0.21 | 0.27 | 0.31 | 0.28 | 0.25 | 0.30 | 0.30 | 0.38 |
| 6－14 | 0.58 | 0.55 | 0.75 | 0.60 | 0.62 | 0.70 | 0.69 | 0.65 | 0.73 | 0.70 | 0.81 |
| 15－19 | 0.71 | 0.73 | 0.68 | 0.48 | 0.64 | 0.78 | 0.80 | 0.79 | 0.78 | 0.80 | 0.87 |
| 20－29 | 0.58 | 0.63 | 0.45 | 0.29 | 0.45 | 0.55 | 0.63 | 0.64 | 0.74 | 0.77 | 0.83 |
| 30－39 | 0.38 | 0.39 | 0.26 | 0.20 | 0.35 | 0.42 | 0.49 | 0.40 | 0.55 | 0.54 | 0.63 |
| 40－59 | 0.31 | 0.29 | 0.28 | 0.18 | 0.27 | 0.42 | 0.43 | 0.38 | 0.48 | 0.48 | 0.52 |
| ≥60 | 0.31 | 0.22 | 0.30 | 0.26 | 0.16 | 0.35 | 0.29 | 0.29 | 0.39 | 0.37 | 0.47 |

A(H3N2)

| **Virus strain** | **A(H3N2)** |  |  |  |  |  |  |  |  |  |  |
| --- | --- | --- | --- | --- | --- | --- | --- | --- | --- | --- | --- |
| Year | **2006** | **2007** | **2008** | **2009** | **2010** | **2011** | **2012** | **2013** | **2014** | **2015** | **2016** |
| Age group |  |  |  |  |  |  |  |  |  |  |  |
| 0－5 | 0.24 | 0.25 | 0.18 | 0.02 | 0.24 | 0.36 | 0.24 | 0.33 | 0.34 | 0.34 | 0.22 |
| 6－14 | 0.50 | 0.56 | 0.45 | 0.03 | 0.46 | 0.59 | 0.53 | 0.76 | 0.81 | 0.63 | 0.72 |
| 15－19 | 0.46 | 0.54 | 0.37 | 0.21 | 0.60 | 0.68 | 0.51 | 0.64 | 0.74 | 0.47 | 0.65 |
| 20－29 | 0.37 | 0.39 | 0.16 | 0.10 | 0.45 | 0.56 | 0.47 | 0.50 | 0.65 | 0.35 | 0.41 |
| 30－39 | 0.23 | 0.27 | 0.09 | 0.09 | 0.33 | 0.51 | 0.44 | 0.49 | 0.57 | 0.44 | 0.42 |
| 40－59 | 0.18 | 0.23 | 0.10 | 0.08 | 0.35 | 0.44 | 0.34 | 0.42 | 0.53 | 0.35 | 0.29 |
| ≥60 | 0.24 | 0.33 | 0.15 | 0.06 | 0.36 | 0.52 | 0.34 | 0.51 | 0.53 | 0.29 | 0.44 |

B/Victoria

| **Virus** | **B/Victoria** |  |  |  |  |  |  |  |  |  |  |
| --- | --- | --- | --- | --- | --- | --- | --- | --- | --- | --- | --- |
| Year | **2006** | **2007** | **2008** | **2009** | **2010** | **2011** | **2012** | **2013** | **2014** | **2015** | **2016** |
| Age group |  |  |  |  |  |  |  |  |  |  |  |
| 0－5 | 0.03 | 0.05 | 0.07 | 0.12 | 0.09 | 0.21 | 0.22 | 0.19 | 0.18 | 0.14 | 0.11 |
| 6－14 | 0.11 | 0.19 | 0.27 | 0.31 | 0.26 | 0.48 | 0.49 | 0.35 | 0.41 | 0.22 | 0.29 |
| 15－19 | 0.08 | 0.20 | 0.31 | 0.27 | 0.29 | 0.57 | 0.50 | 0.40 | 0.40 | 0.20 | 0.32 |
| 20－29 | 0.21 | 0.28 | 0.34 | 0.31 | 0.42 | 0.48 | 0.51 | 0.37 | 0.31 | 0.19 | 0.25 |
| 30－39 | 0.30 | 0.39 | 0.45 | 0.46 | 0.55 | 0.52 | 0.64 | 0.49 | 0.43 | 0.27 | 0.27 |
| 40－59 | 0.10 | 0.15 | 0.21 | 0.32 | 0.39 | 0.49 | 0.52 | 0.44 | 0.39 | 0.24 | 0.29 |
| ≥60 | 0.07 | 0.14 | 0.12 | 0.17 | 0.21 | 0.45 | 0.31 | 0.29 | 0.22 | 0.14 | 0.12 |

B/Yamagata

| **Virus** | **B/Yamagata** |  |  |  |  |  |  |  |  |  |  |
| --- | --- | --- | --- | --- | --- | --- | --- | --- | --- | --- | --- |
| Year | **2006** | **2007** | **2008** | **2009** | **2010** | **2011** | **2012** | **2013** | **2014** | **2015** | **2016** |
| Age group |  |  |  |  |  |  |  |  |  |  |  |
| 0－5 | 0.16 | 0.08 | 0.07 | 0.16 | 0.03 | 0.05 | 0.11 | 0.12 | 0.20 | 0.15 | 0.13 |
| 6－14 | 0.56 | 0.43 | 0.37 | 0.51 | 0.21 | 0.17 | 0.27 | 0.35 | 0.47 | 0.36 | 0.38 |
| 15－19 | 0.80 | 0.65 | 0.71 | 0.78 | 0.51 | 0.38 | 0.48 | 0.58 | 0.60 | 0.46 | 0.52 |
| 20－29 | 0.64 | 0.53 | 0.58 | 0.73 | 0.51 | 0.31 | 0.57 | 0.67 | 0.72 | 0.60 | 0.63 |
| 30－39 | 0.50 | 0.36 | 0.34 | 0.46 | 0.31 | 0.14 | 0.34 | 0.44 | 0.57 | 0.42 | 0.47 |
| 40－59 | 0.34 | 0.25 | 0.27 | 0.44 | 0.25 | 0.16 | 0.26 | 0.39 | 0.41 | 0.34 | 0.34 |
| ≥60 | 0.23 | 0.17 | 0.17 | 0.27 | 0.11 | 0.12 | 0.12 | 0.30 | 0.25 | 0.22 | 0.20 |
